# How Cancer Cells Invade Bladder Epithelium and Form Tumors: The Mouse Bladder Tumor Model as a Model of Tumor Recurrence in Patients

**DOI:** 10.3390/ijms22126328

**Published:** 2021-06-13

**Authors:** Andreja Erman, Urška Kamenšek, Urška Dragin Jerman, Mojca Pavlin, Maja Čemažar, Peter Veranič, Rok Romih

**Affiliations:** 1 Institute of Cell Biology, Faculty of Medicine, University of Ljubljana, SI-1000 Ljubljana, Slovenia; andreja.erman@mf.uni-lj.si (A.E.); urska.dragin@mf.uni-lj.si (U.D.J.); peter.veranic@mf.uni-lj.si (P.V.); 2 Department of Experimental Oncology, Institute of Oncology, SI-1000 Ljubljana, Slovenia; UKamensek@onko-i.si (U.K.); MCemazar@onko-i.si (M.Č.); 3 Institute of Biophysics, Faculty of Medicine, University of Ljubljana, SI-1000 Ljubljana, Slovenia; mojca.pavlin@mf.uni-lj.si; 4 Group for Nano and Biotechnological Applications, Faculty of Electrical Engineering, University of Ljubljana, SI-1000 Ljubljana, Slovenia

**Keywords:** mouse bladder tumor model, bladder tumor, urinary bladder, urothelium, MB49 cancer cells, intercellular junctions, junctional protein

## Abstract

Non-muscle-invasive bladder cancer is the most common form of bladder cancer. The main problem in managing bladder tumors is the high recurrence after the transurethral resection of bladder tumors (TURBT). Our study aimed to examine the fate of intravesically applied cancer cells as the implantation of cancer cells after TURBT is thought to be a cause of tumor recurrence. We established an orthotopic mouse bladder tumor model with MB49-GFP cancer cells and traced them during the first three days to define their location and contacts with normal urothelial cells. Data were obtained by Western blot, immunolabeling, and light and electron microscopy. We showed that within the first two hours, applied cancer cells adhered to the traumatized epithelium by cell projections containing α3β1 integrin on their tips. Cancer cells then migrated through the epithelium and on day 3, they reached the basal lamina or even penetrated it. In established bladder tumors, E-cadherin and desmoplakin 1/2 were shown as feasible immunohistochemical markers of tumor margins based on the immunolabeling of various junctional proteins. Altogether, these results for the first time illustrate cancer cell implantation in vivo mimicking cellular events of tumor recurrence in bladder cancer patients.

## 1. Introduction

Urinary bladder cancer is one of the most common malignancies worldwide and is categorized as non-muscle-invasive bladder cancer (NMIBC) or muscle-invasive bladder cancer (MIBC). Most (75–85%) bladder cancer incidences are non-muscle-invasive and the remaining are either muscle-invasive or metastatic diseases. Despite the transurethral resection of bladder tumor (TURBT) as a standard treatment of NMIC, up to 50% of patients develop tumor recurrence in 5 years and 88% of patients in 15 years [1,2,3]. Recurrence is therefore the main problem for NMIBC patients, making NMIBC a chronic disease with the highest cost per patient of all cancers [4]. Several proposals have been made to explain the high recurrence of bladder tumors. One of them suggests that urothelial cancer cells that have been released from the tumor cell mass during TURBT could subsequently reattach to the traumatized bladder epithelium [5,6]. Recognizing the fate of disseminated cancer cells in the bladder lumen and knowing the very first events of urothelial cancer cell implantation is thus of great importance to understand the mechanisms of recurrence.

Animal tumor models are a useful tool for studies of the development and progression of cancer. The orthotopic rodent tumor models, in which a tumor arises in the orthotopic site, share several characteristics with tumor development in humans and are, therefore, often used for basic and translational research of tumorigenesis and for testing new therapeutic approaches [7,8,9,10]. MB49, a mouse urothelial carcinoma cell line is one of the most frequently used murine bladder carcinoma cell lines. MB49 cancer cells are non-muscle-invasive and non-metastatic urothelial cancer cells [11,12,13,14,15]. In our previous study, we showed that when applied in the bladder, MB49 cancer cells attach to the less differentiated cells of injured urothelium or the exposed basal lamina and form tumors or hyperplastic urothelium [16].

The processes involved in the attachment and implantation of urothelial cancer cells to the urothelium are not clear, and little is known about direct interactions and cell junctions between cancer and normal urothelial cells. Cadherins and catenins are key components of adherens junctions. It is well documented that upregulation of N-cadherin, a mesenchymal marker in epithelial–mesenchymal transition (EMT), and repression of E-cadherin, an epithelial marker, promote migratory phenotype and invasiveness of cancer cells [17,18,19]. The expression of N-cadherin is also closely related to the increased shedding of tumor cells in situ [20]. β-catenin is also involved in the control of cell proliferation, adhesion, and migration. Its upregulation in urothelial cells causes hyperplasia, which could further lead to the development of urothelial carcinoma [21]. The integrins are a family of heterodimeric cellular adhesion glycoproteins involved in cell–cell and cell–extracellular matrix adhesion [22]. In normal tissue, they participate in numerous cell processes, such as cell proliferation, growth, and migration. In cancer, however, they are involved in local invasion, intravasation, and extravasation of cancer cells [23,24]. Specifically, the alpha 3 integrins were shown in normal urothelial tissue as well as in non-invasive and invasive bladder cancers [25,26], whereas the beta 1 integrins are receptors for basal lamina components and participate in metastatic processes [27,28].

Matrix metalloproteinases (MMPs) are key enzymes for the degradation of extracellular matrix components and, as such, promote cancer cell invasion. Moreover, they are involved in epithelial–mesenchymal transition (EMT), cleavage of E-cadherin, and processing of some integrins, which all result in the increased migratory phenotype of cancer cells [29,30].

The main goal of TURBT is the complete removal of bladder tumors [31]. However, in the case of small and occult tumors, it is difficult to accurately determine the depth and exact margins of the tumor. We, therefore, extended the present analysis of cell junctions also to bladder tumors intending to find out if any of examined junctional proteins could serve as a marker of tumor margins.

Our study aimed to determine the fate of urothelial cancer cells in the urinary bladder by tracing them during the first few hours and days after intravesical application. We examined the attachment and implantation of cancer cells by studying intercellular junctions between cancer and normal urothelial cells. We focused on junctional proteins, such as N-cadherin, β-catenin, desmoplakins 1 and 2, and α-integrin, and used various light and electron microscopy techniques to identify the type of cell–cell contacts. Furthermore, we verified whether MB49 cancer cells secreted matrix metalloproteinases (MMPs). Finally, intercellular junctions inside the urothelium of developed tumors and at tumor margins were studied to find potential markers for the precise determination of tumor margins at the microscopic level.

## 2. Results

**Scheme 1:**
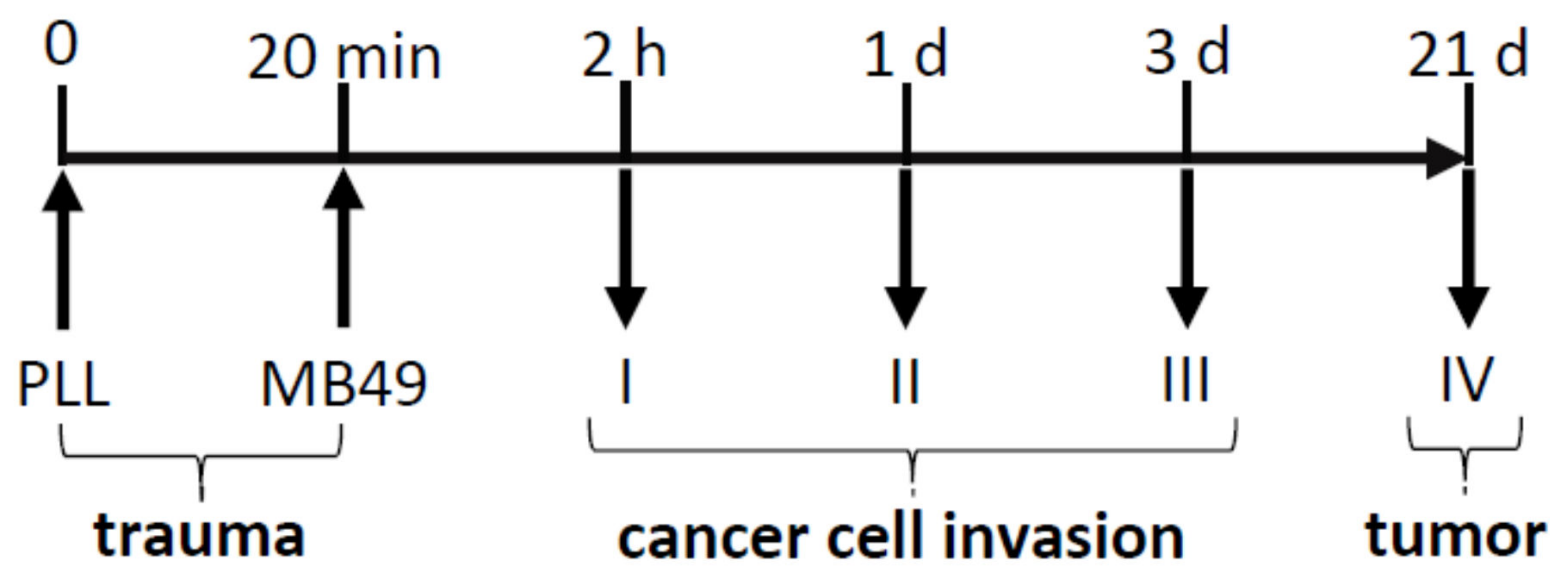
Schematic outline of the experimental design. Traumatization of the urothelium was performed by intravesical treatment with poly-l-lysine (PLL). MB49 cancer cells were then introduced into the bladder lumen and animals were sacrificed after 2 h, 1 day, 3 days, and 3 weeks. Upright arrows demonstrate the introduction of the catheter and downward arrows demonstrate time points of sample collections. Numbers I, II, III, and IV denote animal groups I to IV.

We characterized the early stages of bladder tumorigenesis and investigated established bladder tumors. The experimental workflow is shown in Scheme 1. First, mice were catheterized and the traumatization of the urothelium was performed by intravesical injection of poly-l-lysine (PLL) as previously described 16. Thereafter, MB49-GFP cancer cells with internalized metal nanoparticles were inserted into the mouse bladder. Urinary bladders were collected after 2 h, 1 day, 3 days, and 3 weeks. Results showed that cancer cells attached and then migrated through the urothelium during the first 3 days. At week 3, bladders tumors were formed and analyzed.

### 2.1. Attachment of Urothelial Cancer Cells to Normal Urothelial Cells Is Mediated by Filopodia

**Figure 1:**
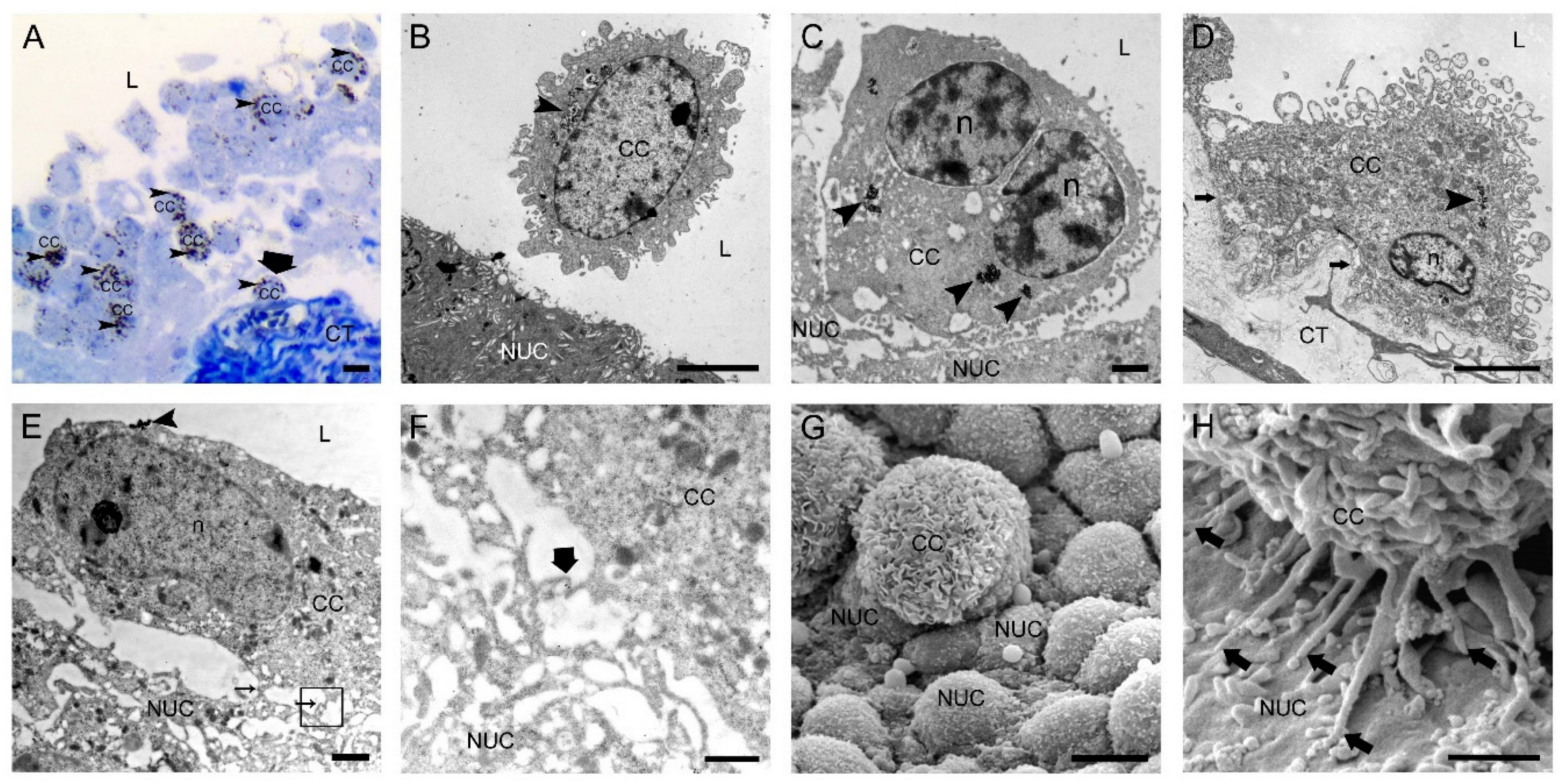
Two hours after intravesical application of MB49-GFP cancer cells with internalized nanoparticles. (**A**) Semi-thin section with numerous floating cancer cells (CC) in the lumen and one cancer cell attached to the exposed basal lamina (thick arrow). Toluidine blue staining. (**B**) Transmission electron micrograph of a floating CC in the lumen and close vicinity to a normal urothelial cell (NUC). (**C**) Transmission electron micrograph of a CC almost adhered to a less differentiated NUC exposed on the urothelial surface due to previously induced desquamation of superficial urothelial cells. (**D**) Transmission electron micrograph of a CC tightly attached to the exposed basal lamina (arrows) due to previously induced urothelial cell desquamation. (**E**) Transmission electron micrograph of a CC with its filopodia (thin arrows) adhered to a NUC at an early step of attachment. The area in the black frame is magnified in F. (**F**) A higher magnification view of the boxed area (black frame) from E with a contact site of thin protrusions of a CC and a NUC. Thick arrow denotes the position of immunogold labeling of α3β1 integrin at the contact site. (**G**) Scanning electron micrograph of a CC attached to the urothelial surface with exposed less differentiated NUCs. Note the typically ruffled plasma membrane of a cancer cell. (**H**) Scanning electron micrograph of a CC attached to a NUC by many tiny filopodia (arrows). Arrowheads (in **A**–**E**)—internalized electron-dense nanoparticles in endosomes as a marker of intravesically applied cancer cells; L—lumen of the urinary bladder; n—nucleus; CT—connective tissue. Bars are 10 µm (**A**,**G**); 5 µm (**B**,**D**); 1 µm (**C**,**E**,**H**); and 600 nm (**F**).

Two hours (animal group I) after intravesical application of MB49-GFP cancer cells with internalized metal nanoparticles, some of them attached to the desquamated areas of the urothelium or the exposed basal lamina. Cancer cells used cell projections—filopodia—with expressed α3β1 integrins on their tips to adhere to less differentiated, newly exposed normal urothelial cells due to previous desquamation of superficial urothelial cells caused by PLL. Internalized metal nanoparticles served as a marker of injected cancer cells under the transmission electron microscope (TEM), while their typical rounded shape and ruffled plasma membrane were confirmed by the scanning electron microscope (SEM) as it was previously described [16] (Figure 1).

### 2.2. Urothelial Cancer Cells Migrate through the Urothelium and Basal Lamina

**Figure 2:**
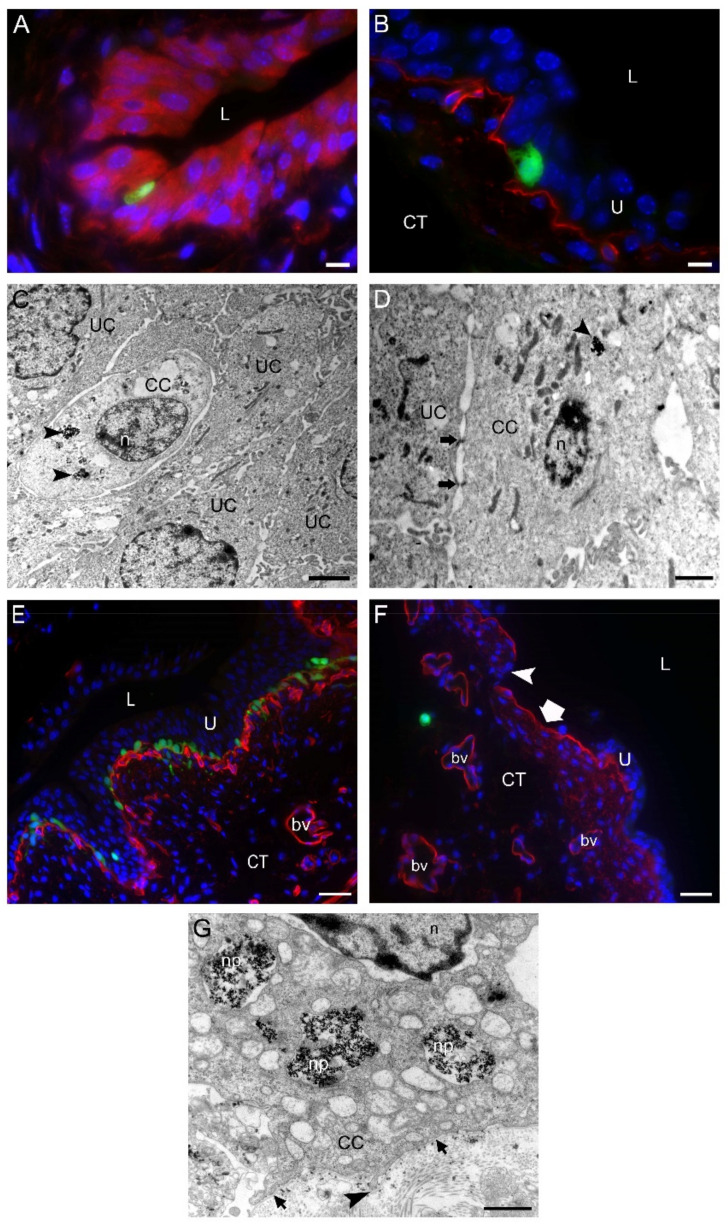
One to three days after intravesical application of MB49-GFP cancer cells (CC) with internalized nanoparticles. (**A**) An MB49-GFP CC (green fluorescence) migrating through the urothelium toward the basal lamina. Urothelial cells were immunolabeled for keratin 7 (red fluorescence). The dark spots in a CC are endosomes with metal nanoparticles. (**B**) An MB49-GFP CC (green fluorescence) reaching the basal lamina immunolabeled for collagen IV (red fluorescence). (**C**,**D**) Transmission electron micrographs of migrating CC with endocytosed nanoparticles (arrowheads). CC during their trans-urothelial migration form desmosomes (arrows in (**D**)) with urothelial cells (UC). (**E**) The region of the urinary bladder wall where numerous MB49-GFP CC (green fluorescence) after the migration through the urothelium are reaching underlying basal lamina immunolabeled for collagen IV (red fluorescence). (**F**) Individual MB49-GFP CC (green fluorescence) penetrating the basal lamina (red fluorescence due to immunofluorescence labeling of collagen IV) and appearing in the connective tissue. Note the area of the urothelium that is completely peeled off (thick arrow), and the part of degraded basal lamina (arrowhead). (**G**) A higher magnification view of the part of the CC with internalized nanoparticles (np) close to the basal lamina (arrows). Arrowhead denotes the invadopodium protruding through the degraded part of the basal lamina. Nuclei were stained with DAPI (blue fluorescence) in (**A**,**B**,**E**,**F**). U—urothelium; L—lumen of the urinary bladder; CT—connective tissue; n—nucleus; bv—blood vessel. Bars are 10 µm (**A**,**B**); 2 µm (**C**), 1 µm (**D**,**G**); and 50 µm (**E**,**F**).

One to three days (animal groups II and III) after intravesical application of MB49-GFP cancer cells with internalized metal nanoparticles, individual cancer cells, or small groups of them, migrated paracellularly through the urothelium toward the basal lamina. Individual desmosomes at contact sites between migrating cancer cells and normal cells inside the urothelium were detected by TEM. On day 3, cancer cells reached the basal lamina. The majority of them were present close to the basal lamina, while individual cancer cells have already penetrated the basal lamina and invaded into the underlying connective tissue. Degraded basal lamina was observed in close vicinity to invadopodia-like protrusions of cancer cells (Figure 2).

### 2.3. Urothelial Cancer Cells Express Junctional Proteins α3β1 Integrin and N-Cadherin In Vitro and In Vivo

**Figure 3:**
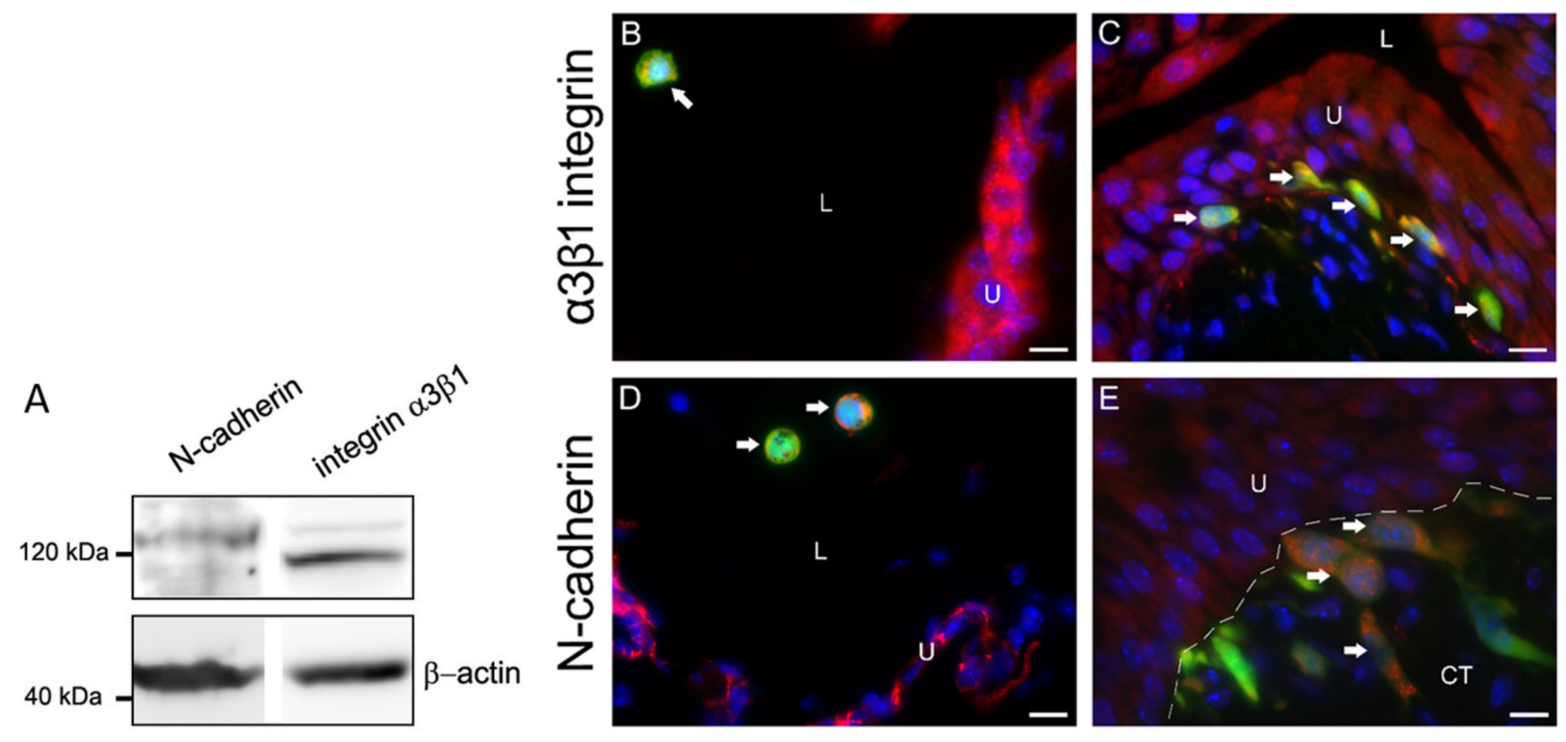
Expression of α3β1 integrin and N-cadherin in MB49-GFP cancer cells with internalized nanoparticles in vitro and in vivo. (**A**) Western blot analysis of MB49 cell culture lysates. The detected bands proved that MB49 cells expressed N-cadherin (120 kDa) and α3β1 integrin (117 kDa). β-actin (45 kDa) was used as a loading control. Molecular weight markers are indicated. (**B**,**D**) GFP-transduced and α3β1 integrin- or N-cadherin-positive cancer cells (arrows) (orange-colored, due to merged green fluorescence of GFP and red fluorescence of α3β1 integrin or N-cadherin) in floating cancer cells (arrows) in the lumen of the bladder 1 hour after intravesical application. (**C**,**E**) GFP-transduced and α3β1 integrin- or N-cadherin-positive cancer cells (arrows) (orange-colored, due to merged green fluorescence of GFP and red fluorescence of α3β1 integrin or N-cadherin) under the basal lamina 72 h after intravesical application. Note dark spots of no fluorescence inside cancer cells, representing internalized nanoparticles in endosomes (in (**B**,**D**)). Nuclei were stained with DAPI (blue fluorescence). The dashed line represents the basal lamina. U—urothelium; L—lumen of the urinary bladder; CT—connective tissue. Bars are 10 µm.

The expression of α3β1 integrin and N-cadherin in cancer cells was constantly present from their attachment to the urothelium or basal lamina at 2 h after intravesical application of cancer cells to their invasion into the connective tissue at day 3 post intravesical application of cancer cells (Figure 3).

### 2.4. Urothelial Cancer Cells Secrete Pro-Forms of MMP-2 and MMP-9

**Figure 4:**
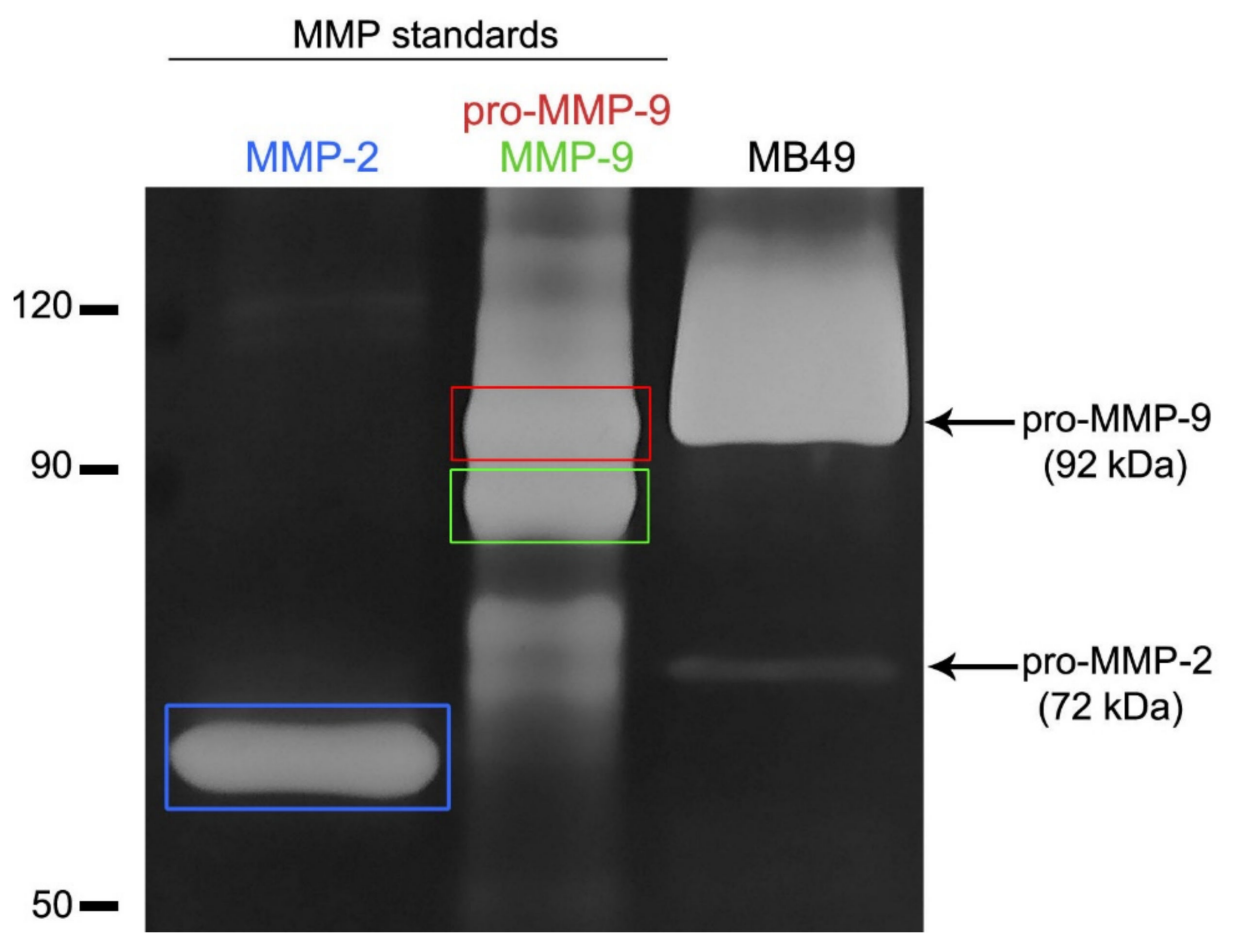
Detection of matrix metalloproteinases (MMP) in the conditioned medium of MB49 cancer cells by gelatin zymography. Gelatinolytic activity was visualized as white bands on dark background. Lanes 1 and 2 indicate recombinant MMP-2 (active form) and MMP-9 (inactive and active form) standard proteins. The detected bands in the conditioned medium of MB49 cancer cells (line 3) showed the presence of pro-form of MMP-2 (pro-MMP-2) and pro-form of MMP-9 (pro-MMP-9).

By gelatin zymography, we have shown that MB49 cancer cells secreted pro-forms of MMP-2 and MMP-9 in the culture medium when cultured in vitro (Figure 4).

### 2.5. Characteristics of Established Orthotopic Bladder Tumors

**Figure 5:**
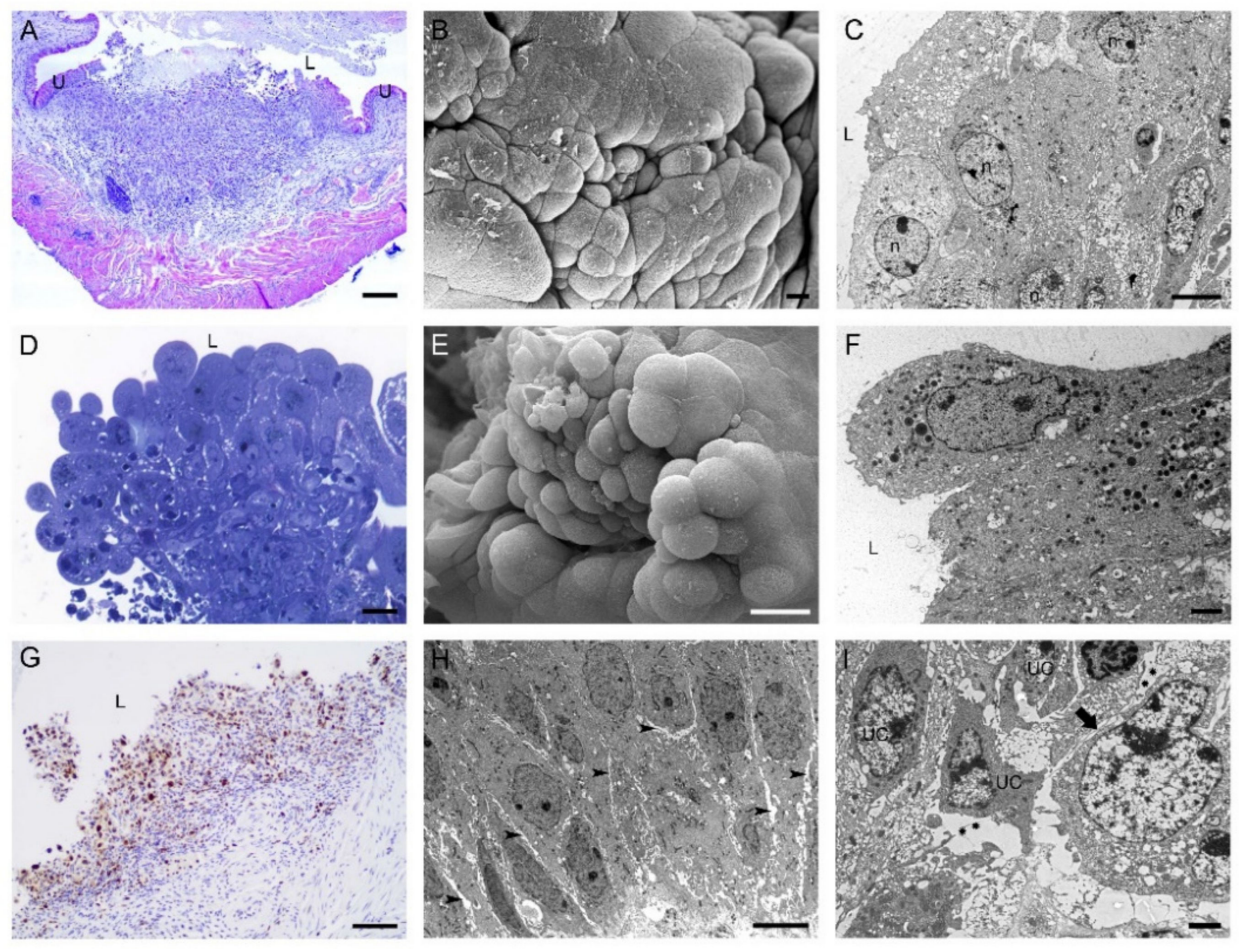
Characteristics of tumors developed in mouse urinary bladders 3 weeks after intravesical application of MB49-GFP cancer cells. Histological and ultrastructural features of sessile bladder tumors in a (**A**) histological section (H&E staining), (**B**) scanning electron micrograph, and (**C**) transmission electron micrograph. Histological and ultrastructural features of papillary tumors in a (**D**) histological section (H&E staining), (**E**) scanning electron micrograph, and (**F**) transmission electron micrograph. (**G**) Bladder tumor with numerous Ki67-positive cells. Nuclei were stained with hematoxylin. (**H**) Ultrastructure of tumor urothelial cells with enlarged intercellular spaces (arrowheads) between them. (**I**) Ultrathin section with the regions of degraded basal lamina (asterisks) under tumor urothelial cells (UC). Arrow denotes an intact part of the basal lamina. U—urothelium; L—lumen of the urinary bladder; n—nucleus. Bars are 200 µm (**A**); 10 µm (**B**); 6 µm (**C**,**H**); 20 µm (**D**,**E**); 2 µm (**F**,**I**); and 100 nm (**G**).

Three weeks (animal group IV) after intravesical application of MB49-GFP cancer cells, one to two tumors per urinary bladder were detected. Tumors of various types and sizes were present on the luminal side of the urinary bladder wall. The majority of them were sessile tumors, while some of them were pedunculated or papillary. All of them showed pronounced nuclear staining of the Ki-67 protein, a marker for the proliferation of tumor cells. Tumor urothelial cells were loosely interconnected with enlarged intercellular spaces between them. On the base of the urothelium, extensive regions of degraded basal lamina were present (Figure 5).

### 2.6. Cell–Cell Junctions between Urothelial Cells of Tumor Formations

**Figure 6:**
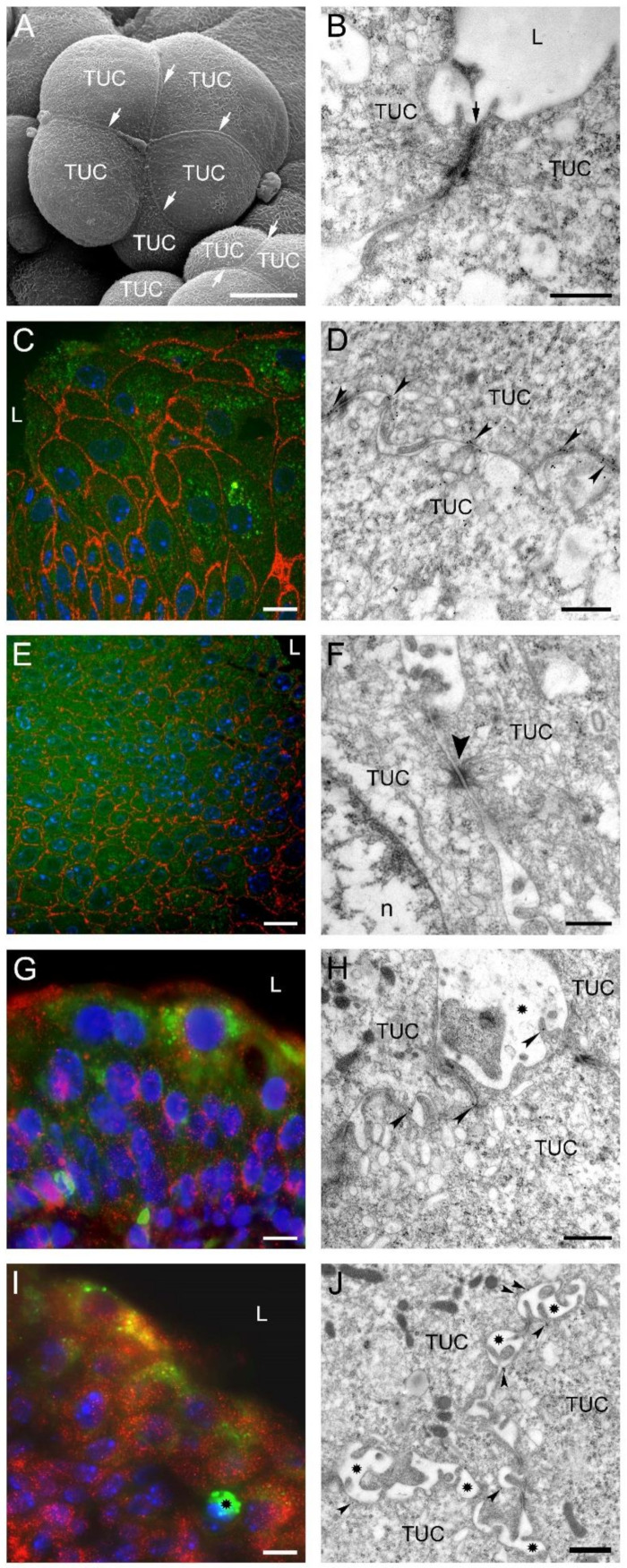
Intercellular junctions between urothelial cells in tumors developed 3 weeks after intravesical application of MB49-GFP cancer cells. Scanning electron micrograph (**A**) and transmission electron micrograph (**B**) of superficial tumor urothelial cells (TUC) connected by well-developed tight junctions (arrows). (**C**) Strong immunofluorescence reaction against β-catenin (red fluorescence) on boundaries of tumor urothelial cells (green fluorescence due to GFP). Nuclei were stained with DAPI. (**D**) Immunogold labeling of β-catenin (arrowheads) on lateral plasma membranes between two adjacent TUC. (**E**) Pronounced immunofluorescence reaction against desmoplakins 1 and 2 (red fluorescence) on boundaries of tumor urothelial cells (green fluorescence due to GFP). Nuclei were stained with DAPI. (**F**) Ultrastructure of two adjacent TUC connected by desmosome (arrowhead). (**G**) Punctate cytoplasmic and membranous immunofluorescence reaction against N-cadherin (red fluorescence) in tumor urothelial cells (green fluorescence due to GFP). Nuclei were stained with DAPI. (**H**) Immunogold labeling of N-cadherin (arrowheads) on lateral plasma membranes between adjacent TUC. Note heavily enlarged intercellular space (asterisk) between cells. (**I**) Strong punctate cytoplasmic immunofluorescence reaction to α3β1 integrin (red fluorescence) in all tumor urothelial cells (green fluorescence due to GFP). Nuclei were stained with DAPI. Asterisk denotes a macrophage. (**J**) Immunogold labeling of α3β1 integrin (arrowheads) on lateral plasma membranes between adjacent TUC. Enlarged intercellular spaces were present between them (asterisks). L—lumen of the urinary bladder; n—nucleus. Bars are 10 µm (**A**,**C**,**E**,**G**,**I**), 600 nm (**B**,**D**,**H**,**J**), 300 nm (**F**).

Superficial tumor urothelial cells were sealed together by prominent tight junctions, observed by transmission and scanning electron microscopes. Immunolabeling of β-catenin and desmoplakins 1 and 2 at the optical and electron microscopy levels revealed the presence of these junctional proteins in the plasma membrane of tumor urothelial cells. β-catenin and desmoplakins 1 and 2 staining was strong in deeper urothelial cell layers but diminishing towards the upper cell layers of the tumor urothelium. Immunofluorescence of N-cadherin and α3β1 integrin was present intracellularly in all tumor urothelial cells, while immunoreaction of both proteins on ultrathin sections was in the plasma membrane or perimembranous and located at contact sites between tumor urothelial cells (Figure 6).

### 2.7. Intercellular Junctions between Tumor Urothelial Cells and Normal Urothelial Cells at Tumor Margins

**Figure 7:**
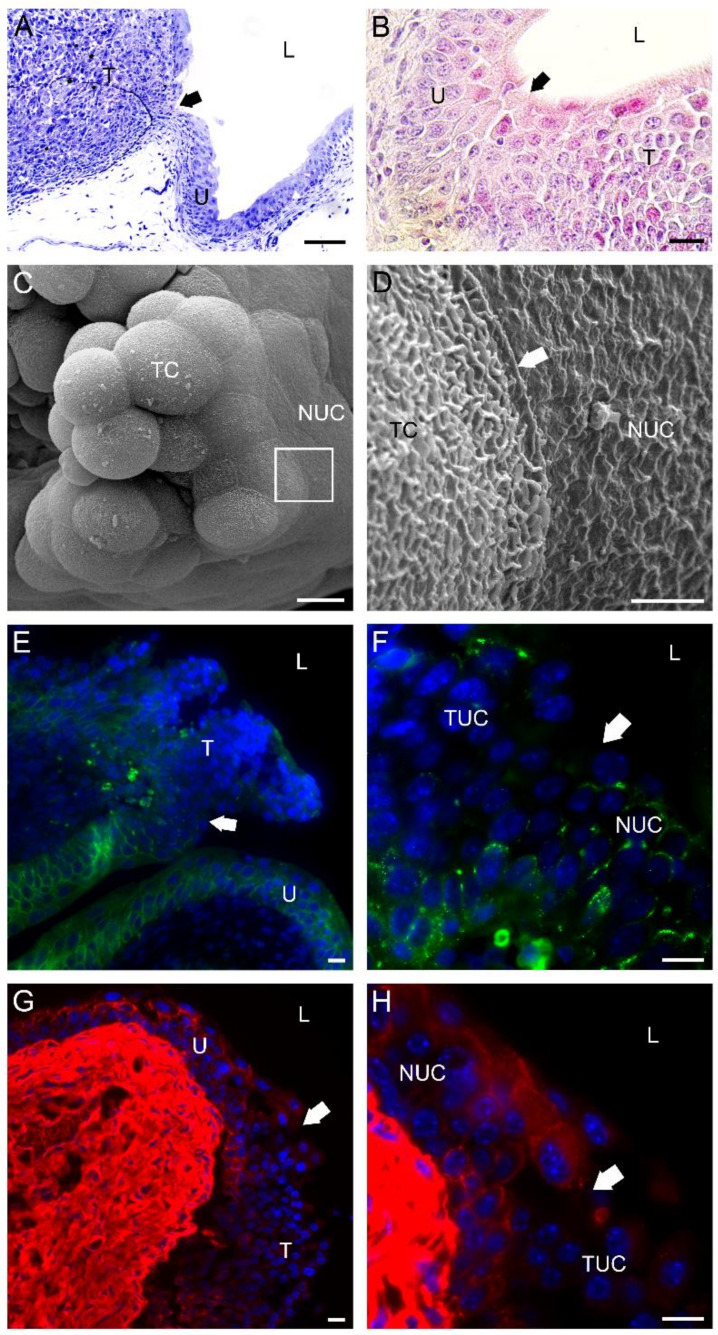
Characteristics of a bladder tumor margin 3 weeks after intravesical application of MB49-GFP cancer cells. (**A**) Semi-thin section of a bladder tumor with an easily recognized margin (arrow) between the urothelium (U) and prominent tumor formation (T). Toluidine blue staining. (**B**) Histological section of bladder tumor with a hardly recognized margin (arrow) between the urothelium (U) and sessile tumor formation (T). H&E staining. (**C**) Scanning electron micrograph of a papillary tumor with an outlined tumor margin (boxed area). (**D**) A higher magnification view of the tumor margin (the boxed area from (**C**)) with well-developed tight junctions (arrow) between a tumor cell (TC) and a normal urothelial cell (NUC). Note the different structures of the plasma membranes of the two cell types. The tumor cell had a typically ruffled plasma membrane, while the urothelial cell had a characteristic scalloped appearance of the apical plasma membrane. (**E**) The tumor margin (arrow) was revealed due to a positive immunofluorescence reaction against E-cadherin (green fluorescence) among cells of the urothelium (U) and a negative immunofluorescence reaction against E-cadherin among urothelial cells of the tumor formation (T). Note that a weak E-cadherin positivity was present only in deeper urothelial layers of the tumor. (**F**) A higher magnification view (from **E**) of the tumor margin (arrow) exposed due to different immunofluorescence patterns of E-cadherin (green fluorescence) in normal NUC than in tumor urothelial cells (TUC). (**G**) The tumor margin (arrow) was revealed due to positive immunofluorescence reaction against desmoplakin 1/2 (red fluorescence) among cells of the urothelium (U) and negative immunofluorescence reaction against desmoplakin 1/2 among cells of tumor formation (T). Note that desmoplakin 1/2 positivity was present only in deeper urothelial layers of the tumor. (**H**) A higher magnification view (from (**G**)) of the tumor margin (arrow) exposed due to different immunofluorescence patterns of desmoplakin 1/2 (red fluorescence) in NUC than in TUC. Nuclei were stained with DAPI (blue fluorescence) in (**E**–**H**). TC—tumor cell; L—lumen of the urinary bladder. Bars are 100 µm (**A**); 20 µm (**B**); 10 µm (**C**,**E**–**H**); and 2 µm (**D**).

The margins of established bladder tumors were not always well defined. Tumor margins of prominent and pedunculated tumors were easily recognized from the luminal side and tumor cells at the boundaries were connected to normal urothelial cells by well-developed tight junctions. In contrast, in sessile tumors, where the margins were unfeatured and hardly recognized, only the immunolabeling of junctional proteins revealed the exact tumor margin due to a different immunoreaction pattern at one side of the tumor margin than at the other. In the case of E-cadherin and desmoplakins 1 and 2, pronounced staining was present in all cell layers of normal urothelium. In contrast, from tumor margin further, where normal urothelium ends, positive staining of both junctional proteins was distributed only in deeper urothelial cell layers of the tumor, while weak or absent immunoreaction was detected in the upper urothelial cell layers of the tumor (Figure 7).

## 3. Discussion

The main problem of patients with a non-muscle-invasive cancer is high recurrence after TURBT. Experimental and clinical data show that bladder tumor recurrence could be due to either intraepithelial expansion of cancer cells from the existing tumor or shedding of cancer cells from the tumor cell mass and their reattachment on traumatized urothelium caused by resection of the tumor [5,32,33]. It is therefore of great importance to understand the initial steps of cancer cell attachment to the urothelium and their further fate inside the bladder. Additionally, elucidation of early events that drive the bladder tumor recurrence may also facilitate new methods for the prevention of tumor cell reimplantation.

The processes of epithelial cancer progression and metastasis with the loss of epithelial identity and the gain of mesenchymal characteristics during epithelial–mesenchymal transition (EMT) are well described, while the formation of direct contacts between cancer cells and normal cells has been neglected. To get more insight into the latter issue, we traced intravesically injected MB49 cancer cells inside the mouse urinary bladder and explored their fate and their contacts with normal urothelial cells. For this reason, we prepared GFP-transduced MB49 cancer cells to observe them inside the bladder by fluorescence microscopy. To identify the MB49 cancer cells with transmission electron microscopy, we labeled the cells with metal nanoparticles, as already published [16]. In addition, at the very beginning of our work on mouse orthotopic bladder tumor, we first had to analyze the MB49 cells on the morphological and molecular level. Despite the comprehensive use of these cells, surprisingly little data about them is available in the literature. Our observation of MB49 cells in vitro by SEM showed that they formed small polyps or tumor-like formations, where MB49 cells were connected by desmosomes, as confirmed by TEM. Furthermore, our comprehensive immunolabeling analysis of protein expression in MB49 cancer cells showed the positivity of several other uroepithelial markers, such as uroplakins, keratins, and tight junctional, epithelial adherence junctional, and desmosomal proteins, but also of some mesenchymal markers, such as N-cadherin, vimentin, and H-ras. Our findings generally proved that the MB49 cancer cells we used are epithelial in nature. There exists a discrepancy between our results and the results of Saito and colleagues describing a considerably more mesenchymal phenotype of the MB49 cells [34]. As the MB49 cell line is in use for a long time and considerable differences can appear in the same cell line by spontaneous mutations, it is not surprising that the discrepancies appear when using different subtypes of the MB49 cells. The variability of cell lines indicates a need for precise characterization of cell lines used as model systems in translational research.

In this study, we performed analyses of a mouse bladder tumor model at three early time-points (2 h, day 1, and day 3 after intravesical application of MB49 cancer cells) to obtain data about the location of cancer cells and their interactions with normal urothelial cells. At the fourth selected time-point (3 weeks after intravesical application of MB49 cancer cells), we analyzed the developed tumors with an emphasis on intercellular junctions inside the urothelium of the tumors and at tumor margins.

During the first 2 h in the bladder, the cancer cells attached to damaged urothelium or to exposed basal lamina due to traumatization with PLL, which was consistent with our previously published results [16]. The present analysis of initial contacts between cancer and normal urothelial cells by immunoelectron microscopy proved that cancer cells attached to normal urothelial cells by filopodia with α3β1 integrins expressed on their tips. α3β1 integrins are distributed throughout the urothelium and are also key receptors for uropathogenic *E. coli* [26,35,36,37]. It was noted that they are usually present on cell projections or membrane ruffles and on initial integrin–matrix contacts at the tips of integrin-containing filopodia of spreading fibroblasts [38]. Based on these data, we assume that interactions between cancer and normal urothelial cells are mediated by filopodia with α3β1 integrins on their tips and without organized cell junctions, similarly to well-described integrin-mediated cell contacts in the process of leukocyte extravasation. After the attachment to undifferentiated superficial cells on the urothelial surface, urothelial cancer cells eventually start a directional trans-urothelial migration toward the basal lamina, which we observed on day 1 after the instillation of cancer cells into the bladder. We found out that the paracellular movement of cancer cells through the urothelium involved direct contact sites by α3β1 integrins and even organized intercellular junctions, particularly desmosomes, between cancer cells and normal urothelial cells. Migrating cancer cells were also N-cadherin positive and thus able to form N-cadherin-mediated adherens junctions with urothelial cells, which might contribute to adhesive events during trans-urothelial migration, as reported for melanoma cells during migration through the cerebral endothelium [39,40].

On day 1 after the instillation of cancer cells into the bladder, the cancer cells reached the basal lamina, whereas on day 3, they were already found in the lamina propria. By gelatin zymography, we detected the pro-forms of matrix metalloproteinases MMP-2 and MMP-9 in the conditioned media of MB49 cancer cells cultured in vitro. We hypothesize that the MB49 cancer cells secrete pro-forms of both MMPs also when instilled into the bladder because we found MB49 cancer cells in close contact with the degraded basal lamina. It is well known that activated MMP-2 and MMP-9 can digest various components of the extracellular matrix, including collagen type IV and laminin-5, key components of the basal lamina [41,42], and thereby cause its disruption.

On day 3 after the instillation of MB49 cancer cells into the bladder, we observed cancer cells in the connective tissue just beneath the urothelium or even in deeper layers of the lamina propria, indicating that the MB49 cells had crossed the basal lamina. There are many reports about α3β1 integrin involvement in MMP-2 activation and cancer cell migration in different types of cancer [43,44,45]. In the light of these reports, it is understandable that in our study, MB49 cancer cells, throughout their migration from the lumen to the connective tissue, expressed α3β1 integrin, suggesting that it might participate not only in cancer cell attachment but also in MMP-2 activation. Finally, significantly increased levels of MMP-2 and MMP-9 were detected in the urine of bladder cancer patients, meaning that both MMPs also serve as bladder tumor-specific markers [46,47].

At week 3 after the intravesical application of cancer cells, tumors of various types and sizes developed in mouse urinary bladders. The majority of them were sessile tumors mimicking the T1 stage in humans and only a few of them were papillary tumors as Ta stage bladder tumor in humans. The majority of patients with T1 and Ta bladder cancer undergo multiple TURBTs to control subsequent tumor development or its recurrence [48]. Therefore, the orthotopic mouse bladder tumor model seems to be an appropriate model not only because it provides reliable data about the fate of cancer cells after attachment to the urothelium but also because it mimics the development of recurrent tumors after TURBT in patients. Bladder tumors in our study were composed of a heterogeneous population of tumor cells with typical cell discohesion due to less frequent cell–cell junctions and, consequently, enlarged intercellular spaces also between urothelial cells, as we showed by immunohistochemistry. As we expected, bladder tumors showed positive N-cadherin and negative E-cadherin expression, which has already been shown as a powerful predictor for tumor recurrence following TURBT in patients [49]. Our examination of cell junctions between tumor urothelial cells indicated that they interconnected by adherens junctions and desmosomes and even by tight junctions at the tumor surface. We have also detected α3β1 integrins in tumor urothelial cells, which was consistent with published data about the role of α3β1 integrins in tumor cell adhesion and tumor growth [50,51]. Concerning cell junctions in human bladder tumors, some studies have reported the presence of junctional proteins, such as E- and N-cadherin, catenins, and connexins, inside the non-invasive bladder cancer [52,53,54,55], but intercellular junctions at bladder tumor margins are understudied. Our analysis of cell junctions at tumor margins revealed that surface tumor urothelial cells formed prominent tight junctions with superficial normal urothelial cells, while in lower tissue layers, the expression of E-cadherin and desmoplakins 1 and 2 was significantly weaker in the tumor than in the adjacent normal tissue. Conceivably, this difference in expression patterns in tumor cells versus normal urothelial cells can help us define the tumor border. This finding is in line with generally accepted data about aberrant expression of E-cadherin and repressed formation of cell junctions in tumorigenesis [56]. Accordingly, E-cadherin and desmoplakins 1 and 2 may serve as molecular markers for discrimination between tumor cells and normal urothelial cells and so enable accurate definition of tumor margin. We believe that this finding could be helpful to histopathologists to define the exact tumor margin of sessile tumors, in particular, due to their hardly recognizable histomorphological margins.

We decided to use immunolabeling for the analysis of cell–cell contacts because this technique offers exact visualization of junctional proteins and circumstantial identification of organized intercellular junctions. By immunofluorescence, we observed the expression and distribution of junctional proteins in observed cells. Yet, only by immunolabeling at the electron microscopy level, we could obtain the precise spatial distribution of these junctional proteins. Moreover, only with this method, we were able to prove that a particular examined junctional protein was located exactly in the plasma membrane or close to it and not elsewhere in the cell. So, in our opinion, immunogold labeling is more specific and trustworthy than immunofluorescence. We believe that this is the reason for the discrepancy in our obtained immunoreactions of N-cadherin and α3β1 integrin at the fluorescence versus electron microscopy levels. To our knowledge, this was the first in vivo study where junctional proteins between urothelial cancer cells, as well as between cancer and normal urothelial cells inside the bladder, were identified by immunoelectron microscopy.

The orthotopic bladder tumor model seems to be an excellent model for tracing cancer cells from their implantation to tumor development and faithfully reflects cellular events of tumor recurrence after TURBT in patients. However, as in all model systems using in vitro cultivated cells, the comparison with the situation in patients always retains a certain degree of uncertainty because of various levels of differentiation at which cancer cell transformation can appear in the tissue and because of multiple genetic and epigenetic changes that may happen during culturing a certain cell line.

Results of this study for the first time illustrated the fate of cancer cells instilled in the urinary bladder in vivo and provided evidence about the expression of junctional proteins in cancer cells during adhesion and migration, as well as in tumors themselves. It is worth elucidating the events from cancer cell attachment to tumor formation and also identifying molecular markers of tumor margins. All the obtained data may contribute to the search for new therapeutic approaches that would aim to reduce tumor recurrence in bladder cancer patients.

## 4. Materials and Methods

### 4.1. Cell Line

The mouse bladder carcinoma cell line MB49 (SCC148; Sigma-Aldrich, St. Louis, MO, USA) was cultured in A-DMEM/F12 (1:1) culture medium supplemented with 5% fetal bovine serum, 4 mM Glutamax (all Gibco, Thermo Fisher Scientific, Waltham, MA, USA), 100 U/mL of penicillin, and 100 mg/mL streptomycin (Thermo Fisher Scientific) in a controlled atmosphere at 37 °C and 5% CO_2_. The absence of Mycoplasma contamination was verified by polymerase chain reaction-based assay, before experimental work.

### 4.2. Establishment of MB49-GFP Stable Cell Line

The MB49-GFP stable cell line was prepared using third-generation lentiviral plasmids: the transfer plasmid pLenti PGK GFP Puro (w509-5) (a gift from Eric Campeau and Paul Kaufman (Addgene plasmid #19070;)) [57] encoding the enhanced green fluorescent protein (eGFP) under the phosphoglycerate kinase (PGK) promoter and the puromycin resistance gene for the selection of transduced cells; packaging plasmids pMDLg/pRRE and pMD2.G (a gift from Didier Trono (Addgene plasmids #12259 and #12251)); and the envelope plasmid pRSV-Rev (a gift from Didier Trono (Addgene plasmid #12253)) [58]. Lentiviral plasmids were propagated in *E. coli* under ampicillin selection and purified using the EndoFree plasmid mega kit (Qiagen, Hilden, Germany).

To generate viral particles, the 293T cells (ATCC CRL-3216TM)—grown in DMEM (Gibco) complete medium in a 10 cm Petri dish to 80% confluence—were transfected with plasmids using Lipofectamine 2000 transfection reagent (Invitrogen, Thermo Fisher Scientific). A mixture (2 mL) containing the plasmids (15 μg of pLenti PGK GFP Puro, 2.4 μg of pMD2.VSVG, 4 μg of pMDLg/pRRE, and 1.8 μg of pMD2.G) and Lipofectamine 2000 in Opti-MEM (Gibco, Thermo Fisher Scientific) was added dropwise to the 293T cells. The next day, the medium was replaced with 5 mL of complete DMEM. Medium contacting viral particles was harvested at 48 h post-transfection and 5 mL of fresh DMEM was added to the cells. The harvested media was stored in a polypropylene tube at 4 °C overnight. At 72 h, the media was harvested again and pooled with the stored media. The pooled media was centrifuged at 500× *g* for 10 min and filtered through a 0.45 μm pore-size filter (Merck Millipore, Burlington, MA, USA) to remove cell debris.

For the transduction, MB49 cancer cells were seeded in DMEM complete medium in a 10 cm Petri dish and grown to 70% confluence. On the day of transduction, the medium was replaced with the medium containing viral particles. After 24 h, the medium was replaced again with a fresh medium, and after 48 h, 3 µg/mL of puromycin (Sigma-Aldrich) was added to the medium for the selection of the transduced cells. After 1 week of culturing under the selective pressure of puromycin (3 µg/mL), the cells were plated on a 96 well plate at a concentration of ~1 cell per well to prepare a monoclonal stable cell line. Cells on the 96 well plate were cultured under the selective pressure of puromycin for additional 2 weeks, when they formed colonies. Wells with one colony per well were inspected using fluorescence microscopy (inverted fluorescence microscope IX70; Olympus, Tokyo, Japan) and two clones with a high and uniform GFP fluorescence intensity were selected for further propagation.

### 4.3. Labeling of MB49-GFP Cancer Cells with Nanoparticles

For the identification of MB49-GFP cancer cells and the discrimination between them and normal urothelial cells under the transmission electron microscope in in vivo experiments, we performed in vitro labeling with cobalt ferrite (CoFe_2_O_4_) nanoparticles as explained in our previous study [16]. Briefly, the day before implantation, MB49-GFP cancer cells from the fifth to seventh passages were exposed for 24 h to cobalt ferrite (CoFe_2_O_4_) nanoparticles coated with polyacrylic acid (provided by the group of Mojca Pavlin, Faculty of Electrical Engineering, University of Ljubljana) at a concentration of 100 µg/mL. Nanoparticles were synthesized and characterized as described by Bregar and colleagues [59]. After 24 h, cells were washed with a fresh cell culture medium. The viability of cells, as determined by a Trypan blue test, was always >96%. MB49-GFP cancer cells at 70–80% confluence were harvested into a single cell suspension by TrypLE Select (Gibco, Thermo Fisher Scientific) and resuspended in A-DMEM/F12 (1:1) medium before injection into animals.

### 4.4. Western Blot Assay

Western blot assay was used to check if MB49 cancer cells expressed junctional proteins N-cadherin and α3β1 integrin. Sub-confluent cultures of MB49 cancer cells were washed and scraped into cold sterile PBS. The cell suspension was centrifuged (10 min, 200× *g*, 4 °C) and pellets were stored at −80 °C. The pellets were thawed on ice and lysed with RIPA buffer for 30 min on ice. Samples were then centrifuged (10 min, 10,000× *g*, 4 °C) and protein concentration in the supernatants was determined by the BCA method (Pierce™ BCA Protein Assay Kit, Thermo Fisher Scientific). Samples were diluted (1:4) with sample buffer supplemented with DTT (Sigma-Aldrich), separated on Novex^TM^ Wedgewell^TM^ 4–20% polyacrylamide gels (Thermo Fisher Scientific) and transferred on nitrocellulose membranes (Amersham Biosciences, Amersham, UK). The membranes were blocked in 5% milk (Pomurske mlekarne, Murska Sobota, Slovenia) in PBS supplemented with 0.1% Tween-20 (T-PBS) (Sigma-Aldrich) for 1 h at room temperature and incubated at 4 °C overnight with polyclonal rabbit antibodies against N-cadherin (1:1000; Abcam, Cambridge, UK), polyclonal rabbit antibodies against α3β1 integrin (1:1000; Abcam), or polyclonal rabbit antibodies against β-actin (1:1000; Sigma-Aldrich), all diluted in T-PBS. The primary antibodies were detected using anti-rabbit polyclonal secondary antibodies conjugated with horseradish peroxidase (Sigma-Aldrich, 1:1000, 1 h, room temperature). Immunoreactivity was visualized using an enhanced chemiluminescence technique (Super Signal West Pico, Thermo Fisher Scientific).

### 4.5. Gelatin Zymography

Gelatin zymography was used to detect the matrix metalloproteinases (MMPs) in the culture medium of MB49 cancer cells. MB49 cells were seeded on the Tissue Culture Flasks (TPP) at a density of 5 × 10^4^ cells/cm^2^, and cultured in the culture medium A-DMEM and F12 (1:1), supplemented with 5% fetal bovine serum, 4 mM Glutamax, and 1% penicillin–streptomycin solution. At a 70–80% confluence, the cell cultures were rinsed with sterile PBS and cultured in the FBS-free culture medium for additional 24 h. Afterward, the culture medium was collected, centrifuged (10 min, 200× *g*, 4 °C), and the supernatants were frozen at −80 °C. Protein concentration in the samples was determined by the BCA method (Thermo Fisher Scientific). The samples with a final concentration of 5 μg proteins/μL were separated on 10% SDS-PAGE gels containing 0.1% gelatin under non-reducing conditions at 4 °C. The recombinant gelatinases MMP-2 (active form, Abcam) and MMP-9 (pro-form and active form, Abcam) with a final concentration of 0.5 ng/μL were loaded as protein standards. After the electrophoresis, the gels were rinsed in distilled water and incubated in the renaturation buffer (2.5% Triton X-100 in distilled water), with gentle agitation, twice in a row for 30 min, at room temperature. The gels were then rinsed with distilled water and incubated in the developing buffer (0.5 M Tris HCl (pH 7.8), 2M NaCl, 0.05 M CaCl_2_, 0.2% Triton X-100 in distilled water) for 16 h at 37 °C. Finally, the gels were stained with Coomassie blue (0.5% Coomassie blue, 5% methanol, 10% acetic acid in distilled water; Bio-Rad, Hercules, CA, USA) for two hours and destained in destaining solution (5% ethanol, 10% acetic acid in distilled water). The MMP activity in the gel was identified by formation of white bands against a blue background.

### 4.6. Animals

Experiments were carried out using 14 to 16-week-old female C57BL/6JOLaHsd mice (Envigo RMS SRL, Milano, Italy). Mice were housed in open-bar cages (2–5 per cage) with bedding (Lignocel ¾, Germany) and enrichment material (paper towel) and under controlled conditions of temperature (22 ± 1 °C), humidity (55 ± 10%), and light (12 h/12 h light–dark cycle with light from 7 a.m. to 7 p.m.) with unlimited access to food (standard rodent diet; Envigo, RMS SRL, Milano, Italy) and water. All procedures involving mice were approved by the National Ethical Committee and the Administration of the Republic of Slovenia for Food Safety, Veterinary and Plant Protection (Permit number 34401-4/2016/8). Animal care and treatment were under Slovenian and international legislation and policy on the protection of animals used for scientific purposes (Directive 2010/63/EU). All animals were monitored daily for body weight and behavior. Animals that were in the experiment for 21 days were additionally monitored daily for clinical status and hematuria by urinary bladder palpation or observation of blood on bedding and white paper towel (enrichment material) and also weekly for consumed food and water.

All mice involved in the study were sacrificed with CO_2_ at the endpoint of each experiment, although untimely euthanasia would be performed according to humane endpoint protocol in the case of >20% body-weight loss, gross hematuria, or signs of pain, which was following the principles of the 3Rs for more humane animal research.

### 4.7. Orthotopic Bladder Tumor Model

Mice were anesthetized with an intraperitoneal injection of ketamine HCl (100 mg/kg; Vetoquinol, Lure, France) and xylazine (10 mg/kg; Chanelle Pharmaceuticals Manufacturing Ltd., Galway, Ireland) and placed in the dorsal position. A polyethylene catheter with a 0.28 mm inner diameter (Intramedic, Becton Dickinson, Franklin Lakes, NJ, USA) was inserted into the bladder through the urethra and sheathed over a 30 G needle connected to a 1 mL injection syringe. The bladder of each animal was emptied by mild manual pressure on the abdomen. A volume of 80 µL of a 0.1 mg/mL poly-l-lysine hydrobromide solution (molecular weight 70–150 kDa) (Sigma-Aldrich) was instilled intravesically to induce cell desquamation in the bladder urothelium. After 20 min, poly-l-lysine was forced out from the bladder by manual pressure on the lower abdomen, and 3 × 10^6^ MB49-GFP cells labeled with nanoparticles, suspended in 80 µL of A-DMEM/F12 medium, were injected for 45–60 min. The catheter with the injection syringe was then removed from the bladder. All infusions were performed gradually and at a slow rate to avoid injury or causing vesicoureteral reflux.

Animals were randomly divided into four groups. Animals of group I (n = 10) were sacrificed 2 h after intravesical injection of cancer cells, animals of group II (n = 10) 1 day after intravesical injection of cancer cells, animals of group III (n = 10) 3 days after intravesical injection of cancer cells and animals of group IV (n = 12) were sacrificed 21 days after intravesical injection of MB49 cancer cells (Scheme 1). For each method, at least three samples of urinary bladders from different animals were analyzed.

### 4.8. Histology and Immunolabeling on Paraffin Sections

For the preparation of paraffin sections of urinary bladders with tumors, the excised urinary bladders were halved and fixed in 10% neutral buffered formalin for 24 h at 4 °C, dehydrated, and embedded in paraffin. Bladders were sectioned into 5 µm-thick tissue sections and stained with hematoxylin and eosin (H&E).

For immunolabeling of the Ki-67 antigen, heat-induced epitope retrieval was performed in a microwave oven for 10 min in citrate buffer (pH 6.0). After cooling, tissue sections were incubated in 3% H_2_O_2_ in methanol for 15 min and rinsed in PBS. Non-specific labeling was blocked by PBS buffer containing 5% FCS and 1% BSA for 1 h at 37 °C. Rabbit monoclonal antibodies against the Ki67 antigen (1:100; Abcam, Cambridge, UK) were applied and incubated overnight at room temperature. After rinsing in PBS, sections were incubated for 1 h in biotinylated swine anti-rabbit antibodies (1:300; Dako, Glostrup, Denmark), followed by incubation in ABC complex/HRP (Dako, Glostrup, Denmark) for 30 min. Negative controls, in which primary antibodies were replaced with 1% BSA in PBS, were also performed. After the standard DAB (Sigma-Aldrich, Germany) development procedure, sections were stained in Mayer’s hematoxylin, dehydrated, and mounted in DePeX (Serva Electrophoresis, Heidelberg, Germany).

### 4.9. Immunofluorescence Labeling on Cryosections and Semi-Thin Cryosections

For the preparation of cryosections, the excised urinary bladders were halved and fixed in 3% paraformaldehyde in PBS for 2 h at 4 °C. After overnight incubation in 30% sucrose at 4 °C, tissue was embedded in Tissue Freezing Medium (Leica Biosystems, Richmond, IL, USA), frozen, and cut in a cryostat chamber (CM3000, Leica) into 5 µm-thick cryosections. Non-specific labeling was blocked by 3% BSA in PBS for 1 h at 37 °C. Primary antibodies (listed in Table 1) were applied and incubated overnight at 4 °C and after rinsing in PBS, the appropriate secondary antibodies were applied for 1 h at 37 °C. Manufacturers of all used antibodies provided proof of validation on the technical specifications. Negative controls, in which primary antibodies were replaced with PBS, were also performed. Sections were mounted in mounting medium Vectashield with DAPI (Vector Laboratories, Maravai LifeSciences, San Diego, CA, USA).

For the preparation of semi-thin cryosections, the excised urinary bladders were cut into pieces of the size of approximately 4 mm^2^, which were placed on microscope slides with the luminal surface facing up and covered by a drop of 3% paraformaldehyde in PBS. The regions with attached MB49-GFP cells were found under the fluorescence microscope Nikon Eclipse TE300 (Nikon, Tokyo, Japan) equipped with a 10× objective and further dissected into small (1 mm^3^) tissue cubes. They were fixed additionally in 3% paraformaldehyde in PBS for 2 h at room temperature, rinsed in PBS, and infiltrated with 12% gelatin. The blocks were further infused with 2.3 M sucrose on a rotator at 4 °C for 2 days, transferred to aluminum pins, and frozen in liquid nitrogen. Semi-thin sections (300 nm-thick) were cut at −100 °C with a cryo-ultramicrotome (UC6/FC6; Leica Microsystems), picked up on a droplet of a 1:1 mixture of 2% methylcellulose/2.3 M sucrose, and mounted on a glass slide. The pick-up solution was removed by washing with PBS. The sections were then incubated with 0.15% glycine in PBS and blocked with 10% (*w*/*v*) BSA in PBS. Sections were incubated in primary antibodies (listed in Table 1) in blocking solution overnight at 4 °C, washed on droplets of 0.1% BSA in PBS, and incubated with appropriate secondary antibodies (goat anti-rabbit-IgG-Alexa 555 or goat anti-mouse-IgG-Alexa 555 for 1.5 h at room temperature. After washing with PBS, sections were embedded in Vectashield with DAPI (Vector Laboratories, Maravai LifeSciences, San Diego, CA, USA). The sections were observed with a fluorescence microscope Nikon Eclipse TE300 (Nikon, Tokyo, Japan).

### 4.10. Bright-Field and Fluorescence Microscopy

Sections were observed and images obtained with a dual bright-field and fluorescence microscope Nikon Eclipse TE300 (Nikon, Tokyo, Japan) with objective lenses (CFI plan-achromat 10x/0.30, CFI plan-achromat 40x/0.60, and CFI plan-achromat 100x/1.3 Oil) and filter cassette with fluorescent filters: UV-2A (Ex: 330–380 nm; Em: 420 nm) for DAPI, B-2A (Ex: 450–490 nm, Em: 520 nm) for Alexa Fluor 488 and G-2A (Ex: 510–560 nm; Em: 590 nm) for Alexa Fluor 555. We used a DS-5M camera (Nikon, Tokio, Japan) to take 2560 × 1920 pixels images and we used NIS-Elements (F3.22.00) as imaging software.

### 4.11. Transmission Electron Microscopy (TEM)

Excised urinary bladders were cut into small pieces and fixed in a mixture of 4% paraformaldehyde and 2% glutaraldehyde in 0.2 M cacodylate buffer (pH 7.3) for 3 h at 4 °C. In short-term experiments (2 h after intravesical injection of MB49 cells), urinary bladders were ligated before the excision to prevent leakage of MB49 cells. Overnight rinsing in 0.33 M sucrose in 0.2 M cacodylate buffer was followed by post-fixation with 1% OsO_4_ for 1 h. After dehydration in an ethanol series, tissue samples were embedded in Epon (Serva Electrophoresis). Epon semi-thin sections (1 µm) were stained with 1% toluidine blue and 2% borate in distilled water for 20 s and observed with a Nikon Eclipse TE bright-field microscope (Nikon, Amsterdam, The Netherlands). Ultrathin sections were contrasted with uranyl acetate and lead citrate and examined in a Philips CM100 transmission electron microscope (Philips, Eindhoven, The Netherlands) at 80 kV.

### 4.12. Immunogold Electron Microscopy (IEM)

Small tissue cubes (1 mm^3^) with attached MB49-GFP cancer cells were localized and isolated as described above for immunofluorescence labeling on semi-thin cryosections. Samples were transferred to 2% paraformaldehyde plus 0.05% glutaraldehyde in PBS for 1 h at room temperature. Samples were dehydrated by progressive lowering of temperature and embedded in Lowicryl^®^ HM20 resin (Polysciences, Warrington, PA, USA) in Leica AFS apparatus by the following protocol: 30% ethanol for 30 min at 0 °C, 55% ethanol for 30 min at −15 °C, 70% ethanol for 30 min at −30 °C, 100% ethanol for 1 h at −50 °C, 75% ethanol/25% HM20 for 1 h at −50 °C, 50% ethanol/50% HM20 for 1 h at −50 °C, 25% ethanol/75% HM20 for 1 h at −50 °C, 100% HM20 for 1 h, 100% HM20 overnight at −50 °C. HM20 was polymerized for 48 h at −50 °C and then for 24 h at −20 °C under UV light. Ultrathin sections (60 nm-thick) were cut and collected on nickel grids. Sections were washed in washing buffer (0.1% Na-azide, 0.8% BSA, and 0.1% IGSS gelatin in PBS), blocked in blocking buffer (5% fetal calf serum in washing buffer) for 30 min at room temperature, and incubated with primary antibodies (listed in Table 1) overnight at 4 °C. After washing in washing buffer, goat anti-rabbit or goat anti-mouse secondary antibodies with 18 nm gold (Au) diluted 1:40 in blocking buffer were applied for 90 min. Sections were counterstained with uranyl acetate and lead citrate. Grids were viewed in a Philips CM100 transmission electron microscope (Philips, Eindhoven, The Netherlands) at 80 kV.

### 4.13. Scanning Electron Microscopy (SEM)

Filled and distended bladders were ligated, excised from animals, cut longitudinally into halves, and fixed for 3–4 h at 4 °C in a mixture of 2% paraformaldehyde and 2% glutaraldehyde in 0.1 M cacodylate buffer (pH 7.4). The tissue samples were rinsed in 0.1 M cacodylate buffer and post-fixed in 1% osmium tetroxide in the same buffer for 1 h at 4 °C. Specimens were critical-point dried, attached on brass or aluminum holders, sputter-coated with gold, and examined in a Tescan Vega3 scanning electron microscope (Tescan, Brno, Czech Republic) at 25 kV.

## Figures and Tables

**Table 1:** Primary antibodies used for immunofluorescence (IF) on cryosections or semi-thin cryosections and immunogold electron microscopy (IEM).

Antibody	Antibody Type	Dilution for IF/ICC	Cat. No.	Manufacturer
Keratin 7	Mouse monoclonal	1:50	MAB3226	Millipore
Collagen IV	Rabbit polyclonal	1:400	Ab6586	Abcam
E-cadherin	Mouse monoclonal	1:100	BD610182	BD Biosciences
N-cadherin	Rabbit polyclonal	1:200/1:100	Ab18203	Abcam
Integrin α3β1	Rabbit polyclonal	1:100/1:50	Ab217145	Abcam
β-catenin	Rabbit polyclonal	1:200/1:100	Ab16051	Abcam
Desmoplakin 1/2	Mouse monoclonal	ready-to-use	65146	Progen
Ki67	Rabbit monoclonal	1:100	Ab16667	Abcam
